# Effect of MHC Haplotype on Mortality Due to Marek’s Disease in Commercial Laying Hens

**DOI:** 10.3390/ani15111647

**Published:** 2025-06-03

**Authors:** Janet E. Fulton, Jesus Arango, Anna Wolc

**Affiliations:** 1 Hy-Line International, Dallas Center, IA 50063, USA; jfulton@hyline.com (J.E.F.); jesus.arango@cobbgenetics.com (J.A.); 2 Cobb Genetics, Siloam Springs, AR 72761, USA; 3 Department of Animal Science, Iowa State University, Ames, IA 50011, USA

**Keywords:** egg layer chickens, Marek’s disease, MHC, genetic resistance

## Abstract

Mortality from Marek’s disease virus (MDV) infection results in economic loss for the poultry industry. It is controlled by vaccination, but the virus mutates and becomes more virulent. Variation within the MHC is well known to impact the outcomes following MDV infection from research performed utilizing the White Leghorn breed, with laboratory strains of the virus. The effect of the MHC haplotype following MDV challenge was determined from six lines of commercial elite (White Plymouth Rock (two), White Leghorn (three), and Rhode Island Red (one)) egg layer lines, challenged with vv+ virus. Mortality was recorded as sire daughter averages at 16–18 weeks of age from 19 generations of data. Sires were genotyped using a set of MHC-specific SNPs, encompassing 210,000 bp. Across all lines, there was a total of 23 unique MHC haplotypes, of which 15 were found at a frequency greater than 5% and used for further analysis. A significant impact on mortality was found for 16 of the haplotypes, with 9 haplotypes associated with decreased mortality and 7 haplotypes with increased mortality. There were three haplotypes identified in more than one line, allowing cross-line comparisons. The effect of these common haplotypes was consistent (either negative, positive or neutral) between lines.

## Simple Summary

Marek’s disease causes significant welfare concerns and contributes to production losses and mortality in unvaccinated flocks. Therefore, identifying genetic variants associated with decreased susceptibility to this disease could benefit both small- and large-scale poultry farmers by increasing inherent resilience following disease outbreaks. The effect of variation within the Major Histocompatibility Complex region of a chicken on survival following Marek’s disease virus challenge (very virulent+ strain, vv+) was examined within six commercially utilized egg production elite lines, including breeds for both white and brown shell eggs. Major Histocompatibility Complex haplotype information was obtained from sires, with mortality from Marek’s disease virus-challenged daughters recorded. Within each line, there were significant differences between haplotypes in total mortality. While not all haplotypes were represented in all lines, where there were common haplotypes between lines, the direction of the effect of the haplotypes was consistent.

## 1. Introduction

Marek’s disease (MD) is a viral disease of chickens caused by the oncogenic herpes virus Marek’s disease virus (MDV) [1,2]. The MDV infection of flocks results in high mortality and immunosuppression, with subsequent decreases in production over the lifetime of a flock. Since the first vaccine was introduced in the 1970s, the virus has mutated with increasing pathogenicity, increasing the severity of the disease, inducing the formation of multiple tumors, neurological disorders, paralysis, and blindness and causing significant mortality and welfare concerns in affected flocks [2,3]. The disease is currently controlled by vaccination, but with the increasing virulence of the virus over time, vaccination is becoming less effective [3,4]. The current vaccines (including the Rispens virus strain) prevent the formation of tumors but do not prevent viral replication [5,6]. This lack of sterilizing immunity likely contributes to the continued increase in pathogenicity observed in the virus [7,8].

The resistance of the host to MDV is of particular importance for the poultry industry as a method to decrease the severity of the disease [9]. Early studies showed that the chicken B blood system was involved with resistance to MDV, with specific B system alleles conferring more resistance than other alleles [10,11]. The B system was determined to be the chicken Major Histocompatibility Complex, a region of the genome containing multiple genes involved with the immune system [12].

Multiple studies have been conducted utilizing a set of inbred MHC-congenic lines. These experimental lines were developed to have an identical inbred genetic background (White Leghorn) but differ at the MHC, thus providing an excellent genetic resource for determining the effect of MHC on disease challenge, without confounding genetic background variables. The seven MHC haplotypes included were common MHC types present in commercial WL birds [13,14,15] as defined by serology. However, these studies have some limitations for relevance in current commercial flocks. The MHC-congenic strains were challenged with relatively mild viruses, as more typical high pathology field viruses were too virulent to allow genetic differences to show. Many of these studies were conducted over 30 years ago, and the virus has increased in virulence since that time. A small study evaluated effects of MHC on Marek’s disease progression with a virulent strain in VALO SPF chickens with inconclusive results [16]. The use of a vv+ strain of the virus is important as this reflects the virulence of the virus that production flocks now encounter in the field [8,9]. Furthermore, there are multiple additional MHC haplotypes present in other breeds (Rhode Island Red and White Plymouth Rock) utilized for commercial egg production [17]. The impact of these non-WL haplotypes on resistance to MDV is unknown, though it has been shown that long-term selection for MD resistance can result in a significant change in MHC haplotype frequency within closed non-inbred lines [9].

Non-MHC genomic regions also impact resistance to MDV, as lines with identical MHC haplotypes but very different MDV responses are known [14]. QTL studies looking for genomic regions that affect MD survival reveal multiple chromosomal regions containing multiple potential candidate genes [18,19,20]. These studies utilizing genome-wide association and genomic prediction analyses show that genomic methods can further enhance reduction in mortality through more accurate selection [18]. A series of recombinant congenic strains developed from the crossing of MD-resistant lines with susceptible lines (MHC identical) showed multiple genomic regions with potential candidate genes that could contribute to resistance to MD [21,22].

The original detection method for B blood system alleles was a hemagglutination assay, which utilized fresh red blood cells and specific alloantisera that differentiated between different alleles. This required the availability of fresh blood samples and the development of highly specific alloantisera for each haplotype [13,23,24,25]. More recently, DNA-based detection methods have been developed which allows retrospective studies utilizing stored DNA with associated Marek’s disease mortality information [9,17,23,26]. Variation in the MHC region is not well captured by SNPs on commercially available SNP chips. To overcome this limitation, an SNP subset from an MHC-specific high-density SNP panel was used to identify all MHC haplotypes present in six commercially utilized elite egg production lines. This 90-SNP panel encompasses 210,000 bp of the chicken MHC region [17]. The previously established identity between serologically defined MHC haplotypes with SNP haplotypes provided information to compare the effect of the same MHC haplotypes under laboratory challenge conditions versus more relevant field conditions and with more varied genetic backgrounds, including WL, WPR and RIR commercially utilized breeds.

The objective of this study was to identify MHC haplotypes present in six commercially utilized elite layer lines, representing the three main breeds utilized for commercial brown and white shell egg production, and evaluate their effect on mortality due to Marek’s disease.

## 2. Materials and Methods

### 2.1. Birds

Samples of DNA were obtained from the Hy-Line International DNA resource library. This library contains DNA from multiple elite lines for all birds used for pedigree reproduction for 26 generations. Information from multiple production traits is associated with each sample, including progeny test-based Marek’s disease mortality. This allowed for a retrospective study to be performed using existing DNA, phenotypic data and DNA-based MHC haplotype detection.

For this study, we used 6 different elite pure egg production lines; White Plymouth Rock (2), White Leghorn (3), and Rhode Island Red (1). The number of males tested per line ranged from 798 to 1127. The mortality data used in this study originated from multiple generations of controlled trials performed between 1997 and 2015 at Hy-Line International to enable the selection of chicken lines for increased resistance to MDV [9]. The data are the mean of approximately 20 to 30 progenies per sire from multiple dams from another line to emulate the crossbred genetics of the commercial production progeny. Day-old pullets were vaccinated with SB-1 Marek’s disease vaccine following standard industry vaccination protocols and then transferred to a brooding house and managed according to standard protocols. SB-1 reduces mortality and improves the welfare of the birds but does not prevent the expression of between-family differences. Pullets were subcutaneously inoculated at 6–7 days of age with 500 viral plaque-forming units of very virulent plus challenge MDV strains (686) provided by the U.S. Department of Agriculture Avian Disease and Oncology Laboratory, East Lansing, Michigan, and transferred to a dedicated, isolated growing house. Standard infectious bursal disease vaccines were also administered following a regular vaccine protocol. Mortality up to 4 weeks of age was recorded as non-MD-related. Mortality was monitored until 17 to 18 wk of age, at which time all survivors were euthanized. In the course of the challenge, birds displaying typical signs of MD (paralysis, blindness, and failure to thrive) were euthanized and visually examined for the presence of tumors. At the end of the test, survivors that showed MD-like symptoms were identified and counted as cases of MD mortality. The treatment of birds met or exceeded the accepted guidelines, and the testing protocol was approved by the Hy-Line animal care committee.

### 2.2. Genotyping

The sires were genotyped with a subset of the SNP panel described by [17] to characterize variation across the chicken MHC. SNPs were genotyped using either single-plex KASP^®^ chemistry [27] or PACE^®^ chemistry (3CR Bioscience Ltd., Harlow, UK). This method uses one common primer and two allele-specific primers and provides SNP genotype information for both alleles. Data from 90 SNPs (from MHCJ6 to MHC178) was used to define the MHC haplotypes present encompassing 210,000 bp of the MHC as previously described [17]. Naming of the haplotypes follows the description provided in [17], in which the BSNP defined haplotype moniker is used as well as the B system serological name (in brackets) when available. All haplotypes found in this study were previously defined by Fulton et al. [17].

### 2.3. Data Analysis

For each sire, the number of copies of a given allele was calculated and fitted in a simple linear model:

Mortality_ijk_ = Test_i_ + βHaplotype_j_ + e_ijk_

where Mortality_ijk_ is the average mortality of the progeny of sire_k_ evaluated in the i-th test (generation) carrying 0, 1 or 2 copies of j-th haplotype and β is the regression coefficient (i.e., the substitution effect).

Regression analysis only evaluates the additive effect of the haplotypes, which is consistent with analysis of progeny data. Each line was analyzed separately, and only haplotypes with a frequency of more than 5% were used. *p* values were adjusted for the 20 tests performed with the false discovery rate (FDR). The analysis was performed using lm and p.adjust functions in R 4.2.1 [28].

The line comparison was performed with a two-way ANOVA fitting effect of the test and line using the lm function in R 4.2.1 [28] with the post hoc Tukey test from the lsmeans package.

## 3. Results

A highly significant impact of the test generation was found. While the challenge conditions, including the facility, specific virus challenge, and method of inoculation, were consistent, several uncontrollable factors such as seasonal and environmental factors, including temperature, humidity, ventilation and diet, were not as easily controlled. The generation of data over 19 years further provides a large degree of uncontrollable variation. Thus, all mortality data included the test/generation effect in the model to adjust for this source of variability. The data show significant differences in MD mortality between lines, ranging from 27% to 45%, with the WL breed showing significantly lower mortality levels than the RIR and WPR breeds (Table 1).

Across all 6 lines, a total of 23 unique haplotypes were identified. In total, 15 haplotypes found at frequencies greater than 5% were used for analysis.

The data were analyzed as the effect of zero, one or two doses of each MHC haplotype (or the allele substitution effect) on Marek’s disease mortality within each line using a regression model, and the results are presented in Table 2. The data with negative values indicate less mortality, thus showing a favorable effect, while the data with a positive value indicate a non-favorable higher mortality. Estimates with a *p* value below 5% are considered significant and the false discovery rate (FDR) provides probability results after correction for multiple tests. Of the 20 analyses performed, within-line significance was found for 15 haplotypes. All haplotype mortality analyses were performed within each line. Within each line, there are highly significant haplotype effects on MD mortality.

## 4. Discussion

Line WL1 contains haplotypes B13 (BSNP-D04(B13)) and B21 (BSNP-A04(B21)). These two haplotypes have been well studied for their relationship with MD mortality in MHC congenic lines, where B13 is considered as a susceptible haplotype, while B21 has been shown multiple times to result in lower MD-associated mortality (resistant) [10,11,13,29,30]. In our study, we also see a significant effect on mortality for these two haplotypes (FDR = 7.48 × 10^−6^) but with opposite ranking as the B13 haplotype shows significantly less mortality than B21; a homozygous B13 sire would result in 4.8% less mortality in his progeny than a B21 homozygous sire. Line WL2 contains three B haplotypes, with B2 (BSNP-K03(B2)) showing a significant increase in mortality (FDR = 2.18 × 10^−67^) relative to the other haplotypes present. A sire homozygous for B2 would result in 20.8% more mortality than sires without the B2 haplotype. This is also different to previously reported results in which B2 is identified as a more resistant haplotype [13,31]. The third WL line (WL3) also contains three haplotypes. BSNP-L01(B15) is identified as associated with a significant increase in mortality compared to the other two present (FDR = 3.68 × 10^−5^). VALO SPF chickens showed a lower number of tumors in B15 birds but a lower number of organs affected with tumors in B21 birds [16]. Previous studies ranking the resistance of MHC haplotypes rank B15 as being a more susceptible haplotype, though this can be dependent on the genetic background [13,32].

Within the WPR1 line, there are three MHC haplotypes with BSNP-M01(B72, B78) showing a significant reduction in mortality of 11.8% (*p* = 2.91 × 10^−31^) compared to the other haplotypes present. Line WPR2 contains five haplotypes, with the most favorable haplotype being BSNP-M01(B72, B78), which provides a 7.1% reduction in mortality for each copy of this haplotype. The other two haplotypes showing significant impact on mortality are BSNP-A09(BQ) and SNP-B03(B22), both of which result in increased mortality. The RIR line contains three haplotypes, with BSNP-M01(B72, B78) having an effect of 3.7% reduced mortality and BSNP-A09(BQ) increasing mortality by 7.4%.

Across all six lines, there are some haplotypes in common. Haplotype BSNP-J06(B12, B71) is found in both WL3 and WPR2; however, in both lines, this haplotype shows no significant impact on %MD mortality. Haplotype BSNP-M01(B72, B78) is found in both of the WPR lines and the RIR line, and in all three lines, this shows as a favorable haplotype, resulting in a significant decrease in mortality in progeny. The third common haplotype, BSNP-A09(BQ), is also found in both of the WPR lines and RIR1. This haplotype presents as unfavorable, consistently resulting in significantly more mortality. The serological BQ haplotype is similar to B21 and is considered to be an MD-resistant haplotype, in contrast to the results observed in this study [33,34,35] in which it shows increased mortality in the three lines where it is present. The presence of common haplotypes between lines provides the replication of the haplotype effect within different genetic backgrounds. All three of these common haplotypes provided consistency in their effect on %MD mortality, regardless of their genetic line.

This study examined the impact on MD mortality of previously unstudied MHC haplotypes found primarily within non-WL breeds. The data clearly show that as with WL haplotypes, there are significant effects of brown eggshell breed MHC haplotypes on survival. This study identifies favorable and unfavorable haplotypes within WPR and RIR breeds. This study also provided an opportunity to compare the impact of previously investigated haplotypes on MD mortality but within very different genetic backgrounds and clearly showed very different results from those previously reported. There are several possible explanations for these differences in favorability. The earlier studies were performed utilizing a set of MHC congenic lines, developed to contain those MHC haplotypes commonly found in commercial WL chickens in the 1970s [14,15]. The background of these lines is the WL inbred strain 15I5, and it is possible that the background genes have a previously unknown impact. The importance of non-MHC genes on resistance to MD is well known [18,19,20]. The earlier studies utilized milder JM11 and MD5 virus strains, and perhaps the vv+ strain used in this study has mutated beyond the protective capacity of B21 [7,30]. Another important factor to consider is that the pathotype of the virus used as the virus-induced mortality ranking of MHC haplotypes can change, depending on the virulence of the challenge virus [31].

While the overall impact of MHC haplotypes on MD mortality reported herein may be considered low, they are both statistically significant and biologically relevant. Any decrease in mortality is considered valuable as it indicates that the bird may be able to respond to infection better and recover quickly, an indication of more resilience under conditions of disease stress. The regression analyses show that specific haplotypes can result in up to 24% less mortality following a challenge (e.g., BSNP-M01 in the WPR1 line), which is a considerable advantage to a flock. This data analysis utilized sire genotypes, with daughter averaged mortality; thus, the progeny of sires homozygous for a favorable allele will have much lower MD-induced mortality.

While this might encourage the fixation of these more favorable alleles in a flock, it is well known that MHC heterozygosity in a flock is extremely important to overall performance [25,36]. Furthermore, while a specific MHC haplotype may be advantageous for one disease challenge situation, it is quite likely that a different MHC may provide an advantage for a different disease, once again supporting the importance of maintaining MHC diversity in chicken flocks. There could be general negative associations for some MHC haplotypes. One example is peripheral neuropathy, which is a neurological syndrome found associated with the B19 haplotype, suggesting that this haplotype is not favorable under some production conditions [35] and perhaps should be removed from commercial flocks. The B19 haplotype has also been shown to be more susceptible to MD in both commercial and laboratory settings [10,11]. Therefore, if increased susceptibility is confirmed to various challenges (viral, bacterial, and parasitic) for a given haplotype, a reduction in its frequency may be an advantageous strategy within a particular genetic background.

## 5. Conclusions

Multiple MHC haplotypes are segregating in commercially utilized chicken lines. Since the final production chicken is produced from multiple line crosses, these birds have high MHC haplotype heterozygosity and diversity, thus providing an advantage under field conditions. While selecting for specific MHC haplotypes can result in reduced mortality due to specific challenges, this also can favor fixation, which can create a risk for overall performance and response to other challenges. Selecting against mortality in general (regardless of the specific haplotype) can also result in reduced mortality due to non-MHC genetic effects. This study confirms the previously determined association of the MHC haplotype with mortality due to MD and adds additional information on previously untested haplotypes from different breeds. However, some of the effects were not consistent with previous inbred-line results, suggesting a potential interaction with background genes or variable responses depending on the viral pathotype.

## Figures and Tables

**Table 1:** Marek’s disease mortality across three breeds (six egg layer lines).

Line	N	Mean % mort.	SD	Tukey Test
WL1	891	28.1	14.52	ab
WL2	1113	29.5	18.25	bc
WL3	1011	27.3	16.97	a
WPR1	798	31.1	16.83	c
WPR2	884	45.3	16.75	e
RIR1	1127	35.2	16.45	d

Rows with the same letter do not differ significantly at alpha = 0.05.

**Table 2:** MHC haplotype frequencies and effects on MD mortality (%) by line.

Line	MHC Type	Frequency	Effect on % MD Mortality ^1^	SE	*p* Value	FDR ^2^
WL1 WL1	BSNP-D04(B13)	0.58	−2.4	0.53	4.49 × 10^−6^	7.48 × 10^−6^
	BSNP-A04(B21)	0.42	2.4	0.53	4.49 × 10^−6^	7.48 × 10^−6^
WL2 WL2	BSNP-E01(B61)	0.30	−6.7	0.66	6.44 × 10^−23^	4.29 × 10^−22^
	BSNP-O03(B10)	0.14	−6.2	0.90	1.18 × 10^−11^	2.95 × 10^−11^
WL2	BSNP-K03(B2)	0.54	10.4	0.55	1.09 × 10^−68^	2.18 × 10^−67^
WL3 WL3	BSNP-V01(B63)	0.07	−5.9	0.93	2.68 × 10^−10^	5.36 × 10^−10^
	BSNP-J06(B12, B71) ^a^	0.42	−0.6	0.47	2.43 × 10^−1^	2.70 × 10^−1^
WL3	BSNP-L01(B15)	0.51	1.9	0.46	2.58 × 10^−5^	3.68 × 10^−5^
WPR1	BSNP-M01(B72, B78) ^b^	0.84	−11.8	0.84	2.91 × 10^−40^	2.91 × 10^−39^
WPR1	BSNP-A09(BQ) ^c^	0.07	9.4	1.35	7.93 × 10^−12^	2.27 × 10^−11^
WPR1	BSNP-A02(B75)	0.08	9.5	1.13	1.77 × 10^−16^	5.90 × 10^−16^
WPR2	BSNP-M01(B72, B78) ^b^	0.15	−7.1	0.81	9.59 × 10^−18^	4.79 × 10^−17^
WPR2	BSNP-Rec21	0.23	−0.2	0.78	7.58 × 10^−1^	7.58 × 10^−1^
WPR2	BSNP-J06(B12, B71) ^a^	0.28	1.1	0.68	1.05 × 10^−1^	1.32 × 10^−1^
WPR2	BSNP-P03(B74)	0.05	1.8	1.39	1.99 × 10^−1^	2.34 × 10^−1^
WPR2	BSNP-A09(BQ) ^c^	0.18	3.6	0.82	1.16 × 10^−5^	1.79 × 10^−5^
WPR2	BSNP-B03(B22)	0.06	3.7	1.30	4.31 × 10^−3^	5.74 × 10^−3^
RIR1	BSNP-M01(B72, B78) ^b^	0.36	−3.7	0.56	1.23 × 10^−10^	2.72 × 10^−10^
RIR1	BSNP-O02(B24)	0.51	−0.4	0.55	5.12 × 10^−1^	5.39 × 10^−1^
RIR1	BSNP-A09(BQ) ^c^	0.11	7.4	0.85	1.24 × 10^−17^	4.97 × 10^−17^

Common haplotypes found in multiple lines are indicated by a, b and c. ^1^ The effect of one additional copy of the haplotype on MD mortality (%). ^2^ False discovery rate values.

## Data Availability

Restrictions apply to the availability of these data. Data were obtained from Hy-Line Int. and are available from the authors with the permission of Hy-Line Int upon a reasonable request.
